# Acid Hydrolyzed Silk Peptide Consumption Improves Anti-Diabetic Symptoms by Potentiating Insulin Secretion and Preventing Gut Microbiome Dysbiosis in Non-Obese Type 2 Diabetic Animals

**DOI:** 10.3390/nu12020311

**Published:** 2020-01-24

**Authors:** Sunmin Park, Ting Zhang, Jing Yi Qiu, Xuangao Wu, Jeong-Yong Lee, Boo-Yong Lee

**Affiliations:** 1 Department of Food & Nutrition, Obesity/Diabetes Center, Hoseo University, Asan 31499, Korea; zhangting92925@gmail.com (T.Z.); yoyo099@163.com (J.Y.Q.); niyani0@naver.com (X.W.); 2 Worldway Co., Ltd., Sanda-gil, Jeonul-myeon, Sejong-si 30003, Korea; dalgoozi@hanmail.net; 3 Department of Food Science and Biotechnology, College of Biomedical Sciences, CHA University, Seongnam 31499, Korea

**Keywords:** silk peptide, gut microbiome, Triglyceride, intestinal cells, glucose tolerance

## Abstract

Silk fibroin hydrolysates have been reported to reduce hyperglycemia, but the mechanism has not been determined in Asian type 2 diabetes (T2DM). We hypothesized that the consumption of acid hydrolyzed silk peptides (SPs) alleviates hyperglycemia by improving insulin sensitivity and subsequently normalizing glucose-stimulated insulin secretion in T2DM. We investigated this hypothesis in a partial pancreatectomized (Px) rat model. Px rats was assigned randomly to the following six groups and fed assigned diet for 8 weeks: the Px-control (0.5 g/kg/day dextrin), the SP-L (0.05 g/kg/day), the SP-M (0.1 g/kg/day), the SP-H (0.5 g/kg/day), the positive-control (40 mg/kg/day metformin), or the normal-control (sham-operated rats; 0.5 g/kg/day dextrin). SPs contained high levels of glycine, alanine, and serine. We found SPs dose-dependently increased food efficiency and body weight gain in Px rats. Animals in the Px-control group rats exhibited lower glucose metabolism, as evidenced by impaired glucose-stimulated insulin secretion coupled with impaired insulin sensitivity, and reduced bone mineral density (BMD) and lean body mass (LBM), compared to the normal-control. SPs and metformin similarly partially protected against Px-induced BMD loss in the lumbar spine and femur. Px-induced decreases in LBM were dose-dependently prevented by SPs, and muscle forces in the SP-M and SP-H groups were maintained at the normal-control level. Glucose tolerance was dose-dependently improved by SPs as determined by oral glucose tolerance and oral maltose tolerance tests, and glucose tolerances were similar in the SP-H and positive-control groups. Insulin tolerance, an index of insulin sensitivity, was dose-dependently enhanced by SPs, and the SP-H group exhibited better insulin tolerance than the positive-control group as determined by intraperitoneal insulin sensitivity testing. Insulin secretory capacity assessed using a hyperglycemic clamp improved in the following order: Px-control <SA-L <SA-M <positive-control <SA-H <normal-control. SP-M prevented gut microbiota dysbiosis. In conclusion, SPs administered at 0.1–0.5 g/kg/day improved glucose regulation by potentiating both insulin secretion and insulin sensitivity in non-obese T2DM rats.

## 1. Introduction

Diabetes prevalence has dramatically increased worldwide due to improved life expectancy and the increased prevalence of obesity. The global prevalence of diabetes was estimated to be 2.8% in 2000 and 8.8% in 2017 in adults aged 20–79 years [1], and its prevalence is higher in low- and middle-income countries than in high-income countries. Type 2 diabetes (T2DM) has markedly increased in Asia in recent years. T2DM is characterized by an imbalance between insulin secretion and resistance. Westerners have a higher capacity to release insulin from pancreatic β-cells to combat high serum glucose levels and not develop T2DM as easily as Asians, and thus, Asians are more susceptible to the disease.

Anti-diabetic medications stimulate insulin-producing pancreatic β-cells and/or improve insulin sensitivity in peripheral tissues, especially in skeletal muscle, adipose tissues, and the liver. The oral hypoglycemic medicines currently used in clinical practice are designed to target insulin resistance and/or glucose-stimulated insulin secretion. However, sulfonylurea and other insulin secretagogues, often exhaust pancreatic β-cells since they act on ATP-sensitive potassium channels to release insulin regardless of serum glucose levels. These drugs are used in Asian T2DM patients that secrete insulin at low levels and immediately reduce serum glucose levels. However, they have various side effects such as hypoglycemia, diarrhea, nausea, and abdominal distension due to gas production and exhausted pancreatic β-cell function. Accordingly, better insulin secretagogues that do not exhaust β-cell mass are needed, as maintaining or increasing pancreatic β-cell mass is important for combating hypoglycemia.

Herbal medicines provide alternative therapeutic options for hyperglycemia, and in particular, mulberry root bark, leaves, and fruits continue to be used to treat T2DM. Furthermore, moranoline and 1-deoxynojirimycin, which are components of mulberry, have hypoglycemic effects [2]. Silk cocoons contain the proteins sericin and fibroin, though fibroin, which mainly contains glycine, alanine, serine, and tyrosine, is the major protein in silk [3]. Interestingly, fibroin has been reported to enhance insulin sensitivity by increasing glucose transport in 3T3-L1 adipocytes [4]. In db/db mice, the consumption of 20% silk fibroin hydrolysates produced using proteases gradually decreased glycosylated hemoglobin levels and increased serum insulin levels [3], which demonstrated silk fibroin hydrolysates have anti-diabetic activities. We hypothesized that the consumption of acid hydrolyzed silk peptides might alleviate hyperglycemia by improving insulin sensitivity and potentiating glucose-stimulated insulin secretion in a non-obese insulin-insufficient partial pancreatectomized (Px) rat model of Asian T2DM.

## 2. Materials and Methods

### 2.1. Acid Hydrolyzed Silk Peptides (SPs)

Dried cocoon hydrolysates of silkworms (*Bombyx mori* L.) were provided by Worldway Co. Ltd. (Sejong, Korea) and stored at −20 °C. Hydrolysates were prepared by washing the cocoons in 13–15 volumes of water and reacting with 2N HCl at 100–110 °C for 12 h. Acid hydrolyzed peptides were filtered and desalted to <0.3% at pH 5.5–7.5. Hydrolysates were then sterilized, concentrated to 20–25 Brix by low-pressure evaporation, and spray dried. Amino acid contents of silk peptides (SPs) were measured using an amino acid analyzer (L-8500; Hitachi, Tokyo, Japan), as described previously [5].

### 2.2. Animals and Ethics

Male Sprague–Dawley rats (218 ± 23 g, aged 8 weeks) were housed individually in stainless steel cages in a controlled environment (23 °C; 12 h light/dark cycle). Rats underwent 90% pancreatectomy using the Hosokawa technique [6] or sham surgery (normal-CON) following anesthesia with ketamine and xylazine (100 and 10 mg/kg body weight injected intramuscularly). Px rats, but not sham rats, developed characteristics of type 2 diabetes (random glucose levels > 180 mg/dL) [6,7]. The Animal Care and Use Review Committee of Hoseo University, Korea, approved all of the surgical and experimental procedures (HSIACUC-19-016(1)).

### 2.3. Experimental Design

Fifty Px rats was assigned randomly to the following five dietary groups: (1) 0.5 g dextrin/kg/day (Px-CON), (2) 0.05 g SPs/kg/day (SP-L), (3) 0.1 g SPs/kg/day (SP-M), (4) 0.5 g SPs/kg/day (SP-H), or (5) 40 mg metformin/kg/day (positive-CON group). Sham-operated rats were given 0.5 g dextrin/kg/day (the normal-CON group; *n* = 10). All rats were allowed free access to water and a high-fat diet (HFD) containing either SPs or dextrin for 8 weeks. The HFD was a modified semi-purified AIN-93 formulation for experimental animals [8] that consisted of 42% carbohydrate, 15% protein, and 43% fat. The major sources of carbohydrate, protein, and fat were starch and sugar, casein, and lard (C.J. Co., Seoul, Korea). Overnight fasted serum glucose levels, food intakes, and body weights were measured weekly. Serum homocysteine concentrations were measured using a Fluorometric Homocysteine Assay Kit (Abcam, Cambridge, MA, USA).

### 2.4. Body Composition Measurements

Body compositions were measured after Px rats were allocated into the group, and they were determined at 7 weeks after study commencement using a dual-energy X-ray Absorptiometer (DEXA; Norland pDEXA Sabre; Norland Medical Systems Inc., Fort Atkinson, WI, USA), which was calibrated using a phantom supplied by the manufacturer. Animals were anesthetized as before with ketamine and xylazine and placed in a prone position with posterior legs at 90° of flexion and maintained in external rotation with tape. Following body scanning, the DEXA unit was set up for measuring bone mineral densities (BMD) in the lumbar spine and femur [9]. Lean body mass (LBM) was measured by DEXA at legs and hips. The results of BMD and LBM were given as the difference between before and after SP treatment for 7 weeks.

Forelimb grip strength was measured using a Grip Strength Meter (GPM-100; Melquest, Toyama, Japan). Briefly, after a rat had grasped the bar mounted on a force gauge and the reading had stabilized, the tail of a rat was slowly pulled back by a researcher. Peak pull force was determined using a digital force transducer in grams [10].

### 2.5. Glucose Homeostasis

Seven weeks into the study an oral glucose tolerance test (OGTT) was conducted in overnight-fasted animals by orally administering 2 g glucose/kg bw [11]. Blood samples were obtained by tail bleeding at 0, 10, 20, 30, 40, 50, 60, 70, 80, 90, and 120 min, and serum insulin levels were measured at 0, 20, 40, 90, and 120 min. Average total areas under the curve (AUC) for serum glucose and insulin levels were calculated using the trapezoidal rule. Three days after OGTT, intraperitoneal insulin tolerance tests (IPITT) were performed 6 h after removing food. Briefly, serum glucose concentrations were determined every 15 min for 90 min after injecting insulin intraperitoneally (0.75 U/kg bw). A Glucose Analyzer II (Beckman-Coulter, Palo Alto, CA, USA) was used for assaying serum glucose, and a rat Ultrasensitive insulin kit (Crystal Chem, Elk Grove Village, IL, USA) was used for the serum insulin determination.

### 2.6. Hyperglycemic Clamp

After 7 weeks of treatment and under ketamine and xylazine anesthesia, the right carotid artery and left jugular vein of 10 animals per group were catheterized. Hyperglycemic clamp testing was conducted while animals were free-moving and after an overnight-fast at 5–6 days post-implantation to measure insulin secretion capacity, as described previously [6,12,13]. Exogenous glucose was infused until serum glucose reached a level of 5.5 mM above baseline, and serum insulin levels were measured 0, 2, 5, 10, 30, 60, and 90 min later. After clamping, rats were provided with food and water ad libitum for 2 days and then deprived of food for 16 h the next day. Rats were anesthetized using ketamine/xylazine and then injected with human insulin (5 U/kg body weight; Humulin; Eli Lilly, Indianapolis, IN, USA) through the inferior vena cava. After 10 min, tissues were surgically excised, flash-frozen in liquid nitrogen, and stored in a cryogenic freezer (−70 °C). Insulin resistance was evaluated using the homeostasis model assessment estimate of insulin resistance (HOMA-IR) calculated as: HOMA-IR = fasting insulin (µIU/mL) × fasting glucose (mM)/22.5. Blood lipid concentrations were assayed using colorimetry kits from Asan Pharmaceutical (Seoul, Korea).

### 2.7. Expression of mRNA in Liver

Total RNA was isolated from liver tissues of five rats per group using Trizol reagent (Life Technologies, Rockville, MD, USA), and 1 μg from each rat was used to synthesize cDNA using a superscript III reverse transcriptase kit (Life Science Technology). Equal amounts of cDNA and primers for specific genes were mixed with SYBR Green mix (Bio-Rad, Richmond, CA, USA) in duplicate and amplified using a real-time PCR instrument (Bio-Rad), as described previously. The peroxisome proliferator-activated receptor (PPAR)-α and PPAR-γ gene primers used were as previously described [13]. Cycle thresholds (CTs) were determined for all samples. Gene expression levels in the liver samples were quantitated using the comparative CT method (ΔΔCT method) [13]. Results were presented as 2^−ΔΔCT^.

### 2.8. Immunohistochemistry

Five rats per group were intraperitoneally injected with BrdU (100 µg/kg body weight) after the 8 weeks treatment period. At 6 h post-injection, rats were anesthetized with ketamine/xylazine and sequentially perfused with saline and a 4% paraformaldehyde solution (pH 7.2). Pancreases were immediately dissected and post-fixed with 4% paraformaldehyde solution overnight at room temperature [14].

To measure β-cell areas, two non-adjacent 5 μm paraffin-embedded tissue sections were selected to avoid counting the same islets twice. BrdU incorporation and apoptosis were assessed by immunohistochemistry, as previously described [14]. Endocrine β-cells were identified by applying guinea pig anti-insulin and rabbit anti-glucagon antibodies to sections. The pancreatic β-cell area was measured by examining all non-overlapping images in two insulin-stained sections from each rat at 10× magnification under a Zeiss Axiovert microscope (Carl Zeiss Microimaging, Thornwood, NY, USA). The mass, individual sizes, proliferation (as determined by BrdU incorporation), and apoptotic cell percentages of pancreatic β-cells were measured as described previously [14]. Pancreatic β-cell mass was calculated by multiplying the pancreatic β-cell area by pancreatic mass.

### 2.9. Next-Generation Sequencing (NGS) of Gut Microbiomes

Gut microbiome compositions of feces were determined by metagenome sequencing using next-generation sequencing (NGS). Bacterial DNA extraction from feces was accomplished using a Power Water DNA Isolation Kit (MoBio, Carlsbad, CA, USA). Each library was prepared using PCR (polymerase chain reaction) products as described by the GS FLX plus library prep guide. emPCR, corresponding to clonal amplification of the purified library, was carried out using the GS-FLX plus emPCR Kit (454 Life Sciences, Branford, CT, USA) [15]. Libraries were immobilized on DNA capture beads, and beads were added to amplification mix and oil and vigorously shaken on a Tissue Lyser II (Qiagen, Valencia, CA, USA) to create “micro-reactors” containing amplification mix and a single bead. Micro-reactor mix emulsions were dispensed into a 96-well plate and amplified by PCR using 16S universal primers in the FastStart High Fidelity PCR System (Roche, Basel, Switzerland) [15,16]. Macrogen (Seoul, Korea) did the DNA sequencing of bacteria from feces using a Genome Sequencer FLX plus (454 Life Sciences).

### 2.10. Statistical Analyses

The significances of differences between the Px-CON, SP-L, SP-M, SP-H, positive-CON, and normal-CON groups were determined by one-way ANOVA. The significance of differences among dietary groups was determined using Tukey’s post-hoc test. Results are presented as means ± standard deviations (SDs). SAS ver. 9.1 (SAS Institute, Cary, NC, USA) was used for statistical calculations, and *p* values of < 0.05 were considered to be significant.

## 3. Results

### 3.1. Amino Acid Composition of SPs

SPs contained high amounts of glycine (8.40 ± 0.43), alanine (7.09 ± 0.51), serine (2.75 ± 0.12), valine (0.71 ± 0.03), and aspartate (0.48 ± 0.02 mg/g powder).

### 3.2. Energy Metabolism and Body Composition

Px-CON rats had much lower body weight gains than normal-CON rats. Body weight gains were reduced dose-dependently by SP treatment, and weight gains in the SP-H group were similar to those of the positive-CON. Food intakes were not significantly different in the six study groups. Food efficiency was lower in the Px-CON group than in the normal-CON group, but similar in the SP-H and normal-CON groups (Table 1). Epididymal fat and retroperitoneal fat masses were much lower in the Px-CON group than in the normal-CON group. Visceral fat mass was also much lower in the Px-CON group than in the normal-CON group, and SPs dose-dependently reduced this difference (Table 1). Visceral fat masses were similar in the SP-H and positive-CON groups.

**Figure 1:**
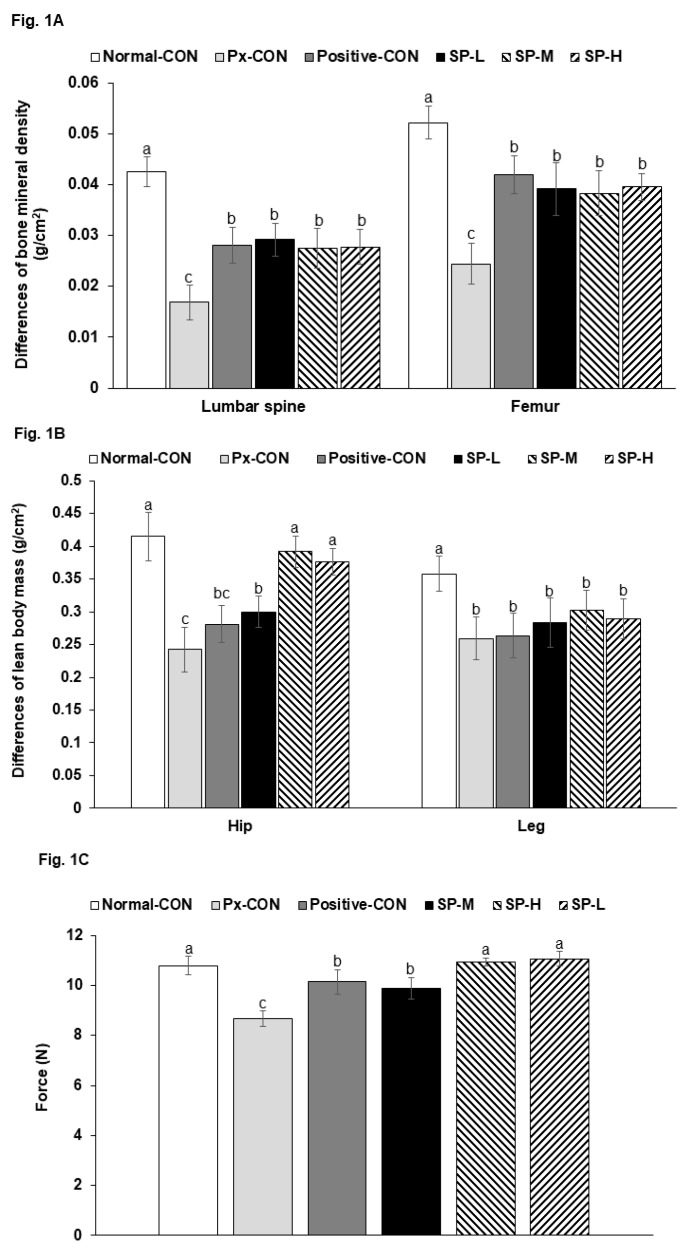
Body compositions and grip strengths. (**A**). The difference in bone mineral densities (BMDs) before and after silk peptide treatments. (**B**). The difference in lean body masses (LBMs) before and after silk peptide treatments. (**C**). Forelimb grip strengths in the 7th week. Pancreatectomized (Px) rats fed (1) 0.5 g dextrin/kg bw (Px-CON), (2) 0.05 g acid hydrolyzed silk peptides(SPs)/kg bw (SP-L), (3) 0.1 g SPs/kg bw (SP-M), (4) 0.5 g SPs/kg bw (SP-H), and (5) 0.04 g metformin/kg bw (positive-CON) with a high fat diet. Sham-operated rats (normal-CON) fed the same diet of Px-CON. Bars and error bars indicate means ± SDs (*n* = 10). ^a,b,c^ Bars with different letters are significantly different (*p* < 0.05).

In lumbar spines and femurs, the reduction in BMD during treatment was much greater in the Px-CON group than in the normal-CON group, and these Px-induced BMD reductions were similarly prevented by SP-H and metformin (positive-CON; Figure 1A). Treatment effects on LBM reduction showed similar patterns. LBM reduction was greater in the positive-CON group than in the normal-CON group, and SPs significantly and dose-dependently protected against LBM loss at hips, but no legs. Metformin did not protect animals from Px-induced LBM loss (Figure 1B). When pulling the tail by a certain force, the force to grasp the bar by the forelimbs was lower in the Px-Con than the Normal-CON, and SPs increased the force. Positive-CON increased the force more than Px-CON and SP-M, and SP-H had a similar force of the Normal-CON (Figure 1C). Thus, SPs protected against Px-induced decline of the force indicating muscle function and LBM loss.

### 3.3. Glucose Metabolism

Overnight-fasting serum glucose concentrations after the 8 weeks treatment period were higher in the Px-CON group than in the normal-CON group, and these increases were dose-dependently lowered by SPs. Serum glucose concentrations at 2 h post-prandial were much higher in the Px-CON group than in the normal-CON group, and SPs also dose-dependently lowered these increases (Table 2). The SP-H and positive-CON group had similar serum glucose concentrations (Table 2). Overnight serum insulin levels were lower in the Px-CON group than in the normal-CON group.

**Figure 2:**
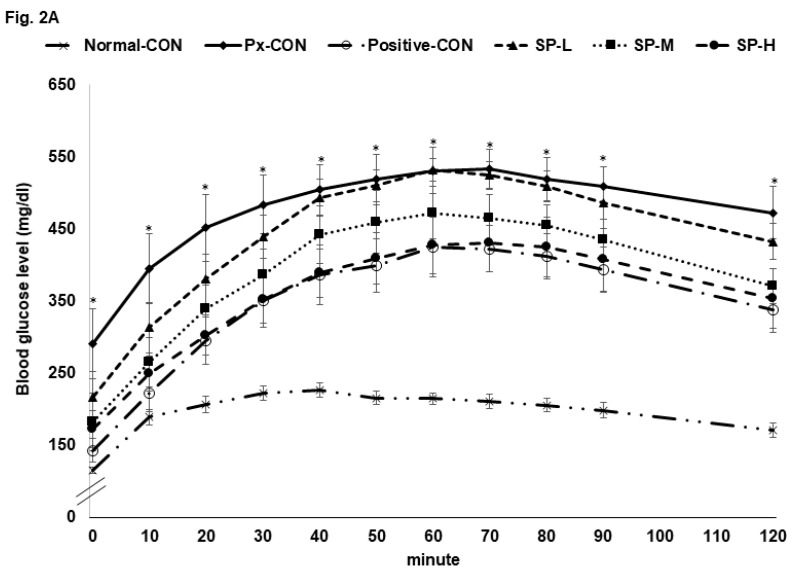

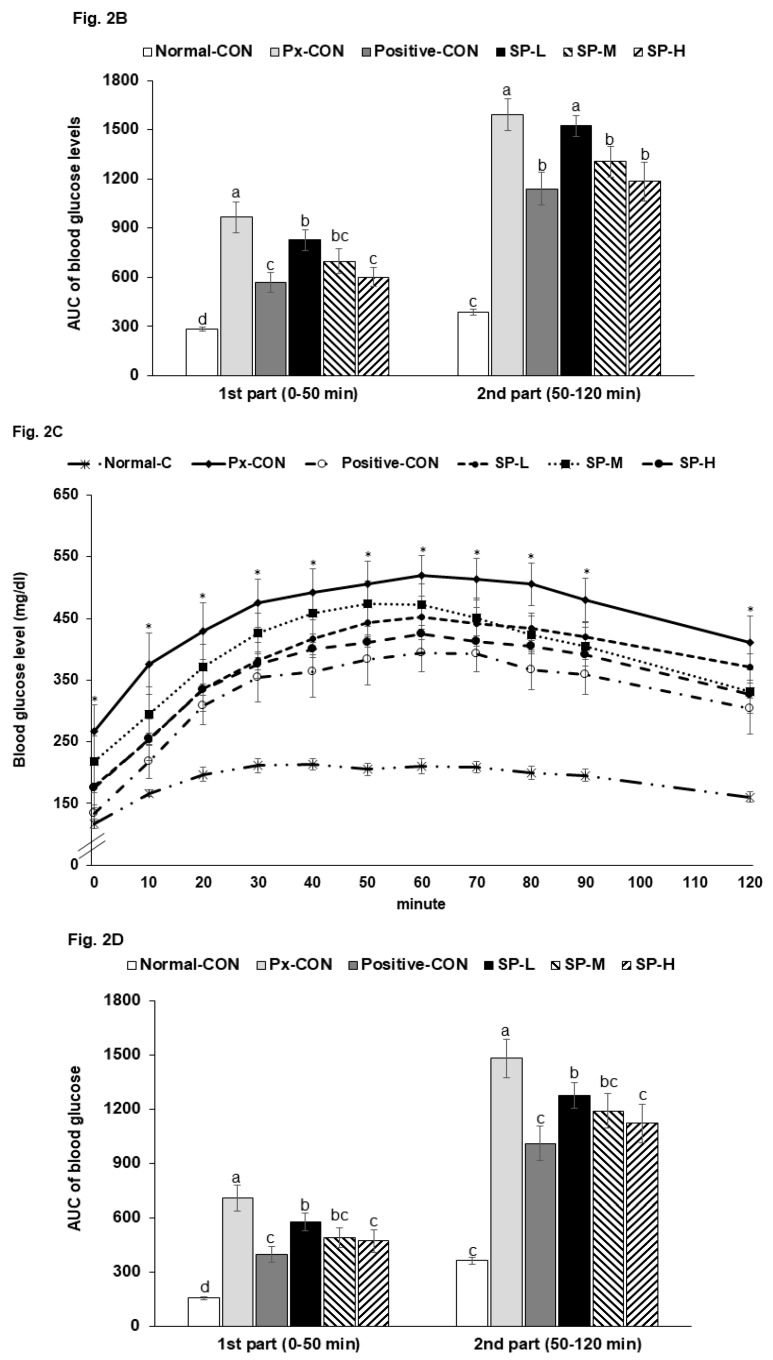
Serum glucose levels during oral glucose tolerance test (OGTT) and oral maltose tolerance test (OMTT) after a 16 h fast. (**A**). Changes in serum glucose concentrations after an oral challenge of 2 g glucose/kg. (**B**). Areas under the curve (AUC) for serum glucose during the first (0–40 min) and second phases (40–120 min) of OGTT. (**C**). Changes in serum glucose concentrations after an oral challenge with 2 g maltose/kg. (**D**). Areas under the curve (AUC) for serum glucose during the first (0–40 min) and second phases (40–120 min) of OMTT. Bars or dots and error bars represent means ± SDs (*n* = 10). * indicates a significant intergroup difference (*p* < 0.05). ^a,b,c^ Different letters on bars indicate significant differences (*p* < 0.05).

The Px-CON group exhibited impaired glucose tolerance after consuming 2 g glucose/kg bw (OGTT) as compared to normal-CON group, and this impairment was alleviated by metformin (Figure 2A). SPs dose-dependently improved glucose tolerance, and glucose tolerance was similar in the SP-H and positive-CON groups (Figure 2A). The serum glucose level AUC during the first phase of OGTT was much higher in the Px-CON group than in the normal-CON group, whereas SPs decreased the serum glucose AUC as compared with the normal-CON group during the 1st and 2nd phases; AUCs of the 1st and 2nd phases in the SP-H and positive-CON groups were similar (Figure 2B).

An oral maltose tolerance test (OMTT) was also conducted to investigate effects of treatments on the digestion of the α-1,4-glycosidic bond and glucose absorption. The changes of serum glucose concentrations in OGTT and OMTT results were similar. SP-L had a greater effect on OMTT than OGTT, whereas SP-M and SP-H had similar effects on both.

**Figure 3:**
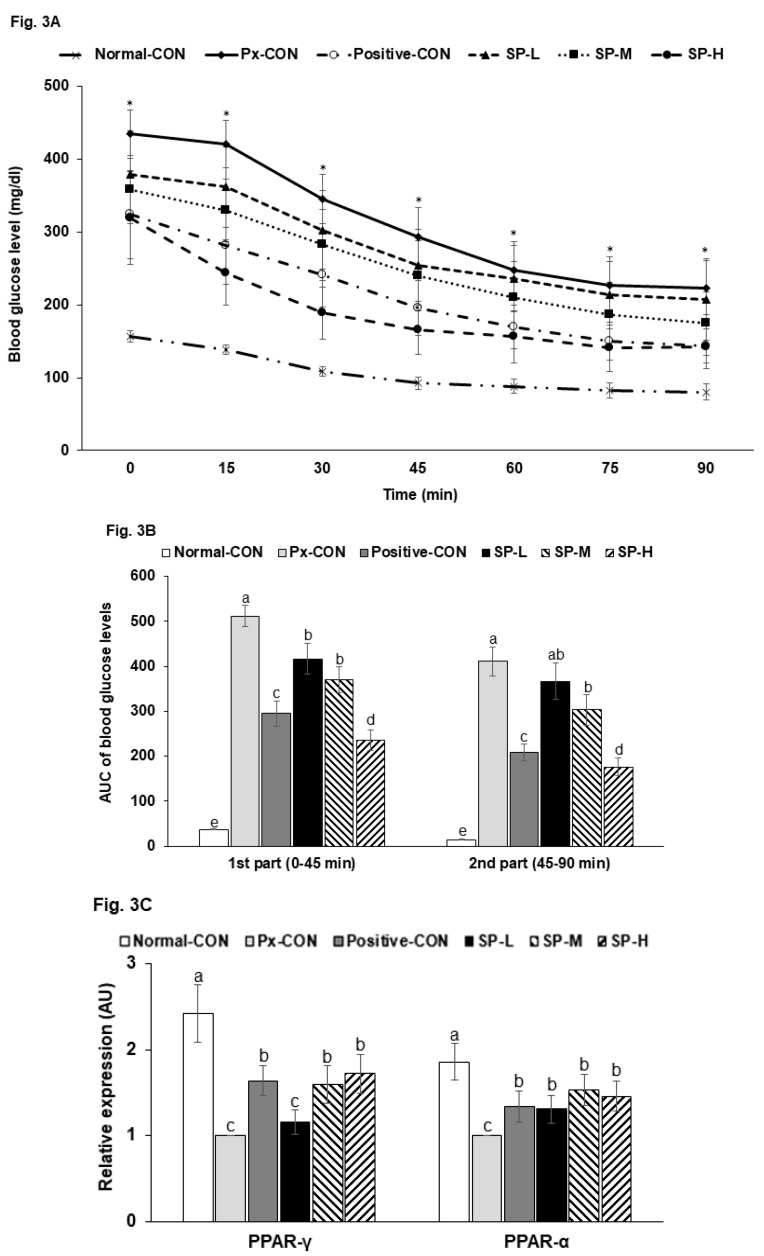
Serum glucose levels during intraperitoneal insulin tolerance testing (IPITT) after a 6 h fast. (**A**). Changes in serum glucose concentrations after an intraperitoneal injection of 1 IU of insulin/kg. (**B**). Area under the curve (AUC) for serum glucose concentrations during the first (0–30 min) and second phases (30–90 min) of IPITT. (**C**). Relative mRNA expression of PPAR-α and PPAR-γ in the liver. Bars or dots and error bars represent means ± SDs (*n* = 10). * indicates a significant intergroup difference (*p* < 0.05). ^a,b,c^ Different letters on bars indicate significant differences (*p* < 0.05).

After 6 h food removal, serum glucose levels were highly elevated in Px-CON compared to the normal-CON group, and mean group glucose levels followed the descending order: Px-CON, SP-L, SP-M, positive-CON, and SP-H (Figure 3A). After intraperitoneal insulin injections, serum glucose levels reduced over 60 min in all groups and then maintained. The AUCs of serum glucose levels in the 1st and 2nd phases of IPITT were much greater in the Px-CON group than in the normal-CON group. SPs dose-dependently decreased serum glucose levels during the 1st and 2nd phases, and SP-H lowered the AUC of both phases more than the positive-CON level (Figure 3B).

Insulin sensitivity was found to be associated with the expressions of hepatic PPAR-α and PPAR-γ (Figure 3C). The mRNA expressions of PPAR-γ and PPAR-α were lower in Px-CON than in the normal-CON, and SP-M and SP-H prevent these reductions as much as positive-CON (Figure 3C).

### 3.4. Insulin Secretion by Hyperglycemic Clamp Test

**Figure 4:**
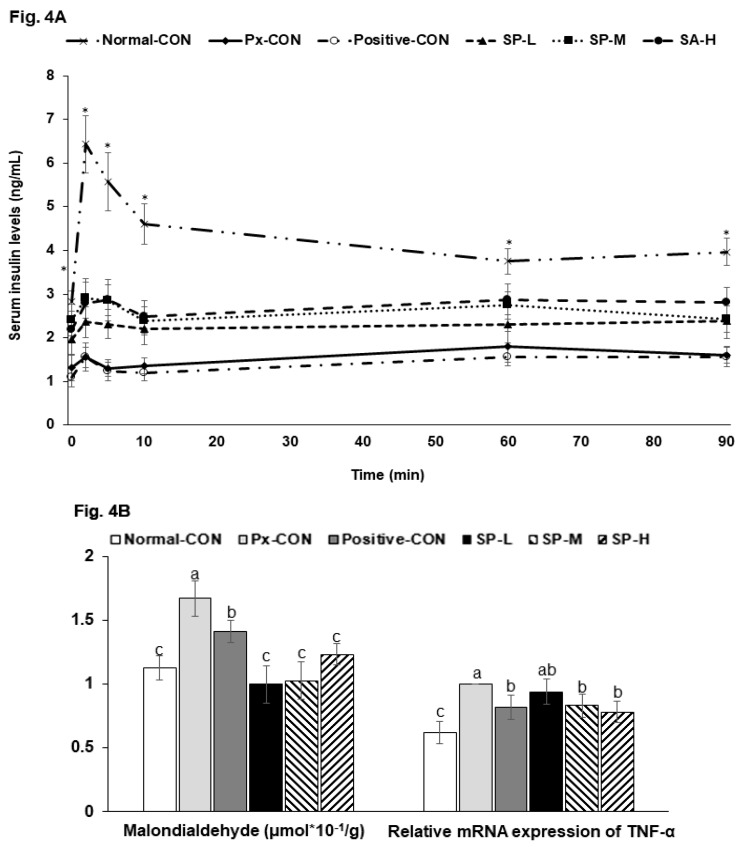
Changes in serum insulin concentrations during hyperglycemic clamp test and malondialdehyde contents and TNF-α mRNA expression in islets. (**A**). Changes of serum insulin concentration during hyperglycemic clamp procedure. (**B**). Malondialdehyde contents and TNF-α mRNA expression in islets. Serum insulin concentrations were determined after glucose infusion to achieve a serum glucose concentration of 100 mg/dL above baseline. Dots and error bars represent means ± SDs (*n* = 10). ^a,b,c^ Different letters on bars indicate significant differences (*p* < 0.05). * indicates a significant intergroup difference (*p* < 0.05).

Fasting serum glucose concentrations were much higher in the Px-CON group than in the normal-CON, and SPs dose-dependently lowered glucose levels (Table 2). At 2 h post-prandial, serum glucose concentrations were elevated to >200 mg/dL in the Px-CON group, and SPs dose-dependently suppressed these increases. Serum insulin levels in the fasted state were lower in the Px-CON group than in the normal-CON group (Table 2). During hyperglycemic clamp testing, serum insulin levels showed an acute phase (0–10 min) and 2nd (60–90 min) phase in all six groups (Figure 4A). The acute phase showed a sharp large peak in the normal-CON rats but a less sharp, smaller peak in Px-CON rats. SPs at all dosages increased in peak height during the acute phase versus Px-CON rats (Figure 4A). In the 2nd phase, serum insulin levels were lower in the Px-CON group than in the normal-CON group. However, the 2nd phase of serum insulin concentrations were higher in SP compared to the Px-CON group (Figure 4A).

The Px-CON and positive-CON groups had similar serum insulin levels during the hyperglycemic clamp test (Figure 4A). Glucose infusion rates required to maintain serum glucose levels at 100 mg/dL above fasting state levels were much lower in the Px-CON group than the normal-CON group (Table 2). SP-H increased glucose infusion rates to the positive-CON level and had a greater effect than metformin. Insulin sensitivity in the hyperglycemic state (60–90 min) was markedly lower in the Px-CON group than the normal-CON group. SP-H increased insulin-sensitivity, but not to the same extent as metformin. Serum homocysteine was much higher in Px-CON group than in the normal-CON group, and SPs dose-dependently reduced Px-induced increases (Table 2).

### 3.5. Pancreatic β-Cell Mass, Proliferation, and Apoptosis

Pancreatic β-cell areas were determined by the number and individual size of β-cells. Increased numbers of β-cells (indicating hyperplasia) suggests an improvement in diabetic status and increases in β-cell sizes (indicating β-cell hypertrophy) reflect increased insulin resistance. Mean β-cell area was greater in the Px-CON group than the in normal-CON group (Table 3), and SPs dose-dependently reduced this increase. Absolute β-cell mass was ~3-fold less in the Px-CON group than in the normal-CON group, and SPs dose-dependently reduced this Px-induced reduction in β-cell mass. Pancreatic β-cell masses in the SP-M, SP-H, and positive-CON groups were similar (Table 3).

The β-cell numbers are dependent on rates of proliferation and apoptosis. β-Cell apoptosis levels were higher and β-cell proliferation was lower in the Px-CON group than in the normal-CON group (Table 3), and β-cell proliferation was lower in the Px-CON group than in the normal and positive-CON groups. β-Cell proliferation was higher in the SP-M and SP-H groups than in the Px-CON group, and higher in the SP-M and SP-H groups than in the positive-CON group. β-Cell apoptosis was greater in the Px-CON group than in the normal or positive-CON groups. SPs decreased Px-induced β-cell apoptosis and were lower in the SP-M group than in the positive-CON group (Table 3).

Apoptosis is associated higher concentrations of malondialdehyde, an index of oxidative stress, and were higher in the Px-CON than in the normal-CON. SPs reduced malondialdehyde levels dose-dependently (Figure 4B). Furthermore, the mRNA expression of TNF-α (a marker of inflammation) was higher in the islets of Px-CON rats than in those of normal-CON rats, and SPs dose-dependently reduced Px-induced increases in TNF-α mRNA (Figure 4B).

### 3.6. Microbiome in the Cecum

**Figure 5:**
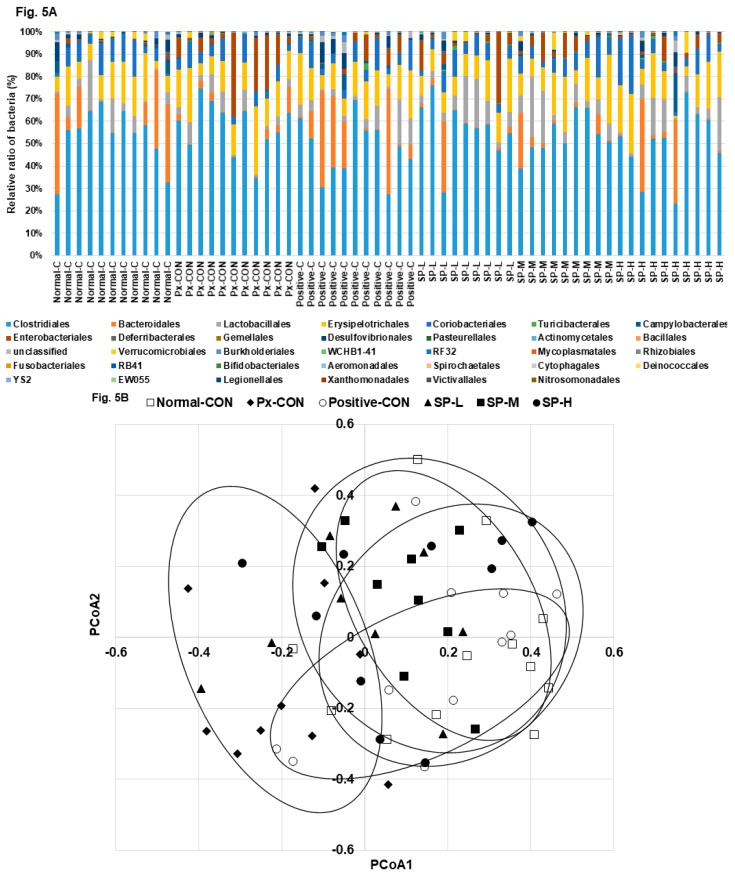

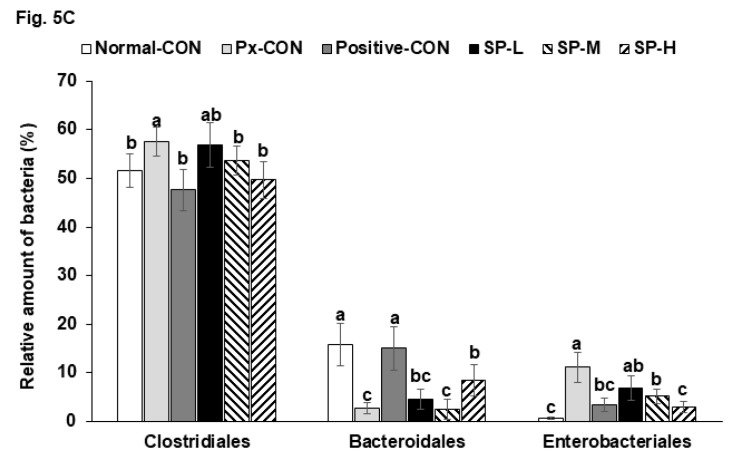
Gut microbiome and area. (**A**). The composition of gut microbiome in the order level. (**B**). The PCoA analysis. (**C**). Relative counts of *Bacteroidales, Clostidales,* and Enterobacteriales based on total bacteria counts. Dots and error bars represent means ± SD (*n* = 8). ^a,b,c^ Different letters on bars indicate significant differences (*p* < 0.05).

The bacterial distributions in the cecum are shown at the order level of bacterial taxonomy in Figure 5A. The most abundant bacteria from high to low concentration were: *Clostridiales*, *Bacteroidales*, *Lactobacillales*, *Erysipelotrichales*, *Enterobacteriales*, *Desulfovibrionales*, and *Verrucomicrobiales* (Figure 5A). Px-CON exhibited a different pattern of gut microbiota compared to the normal-CON, and Positive-CON was different from Px-CON in the order level of the bacteria (Figure 5A). SP-M and SP-H showed a similar pattern of bacteria with the normal-CON and positive-CON in the order level (Figure 5A). Principal coordinate analysis (PCoA) indicated clustering of bacterial communities (Figure 5B). PCoA Px-CON had a separate clustering with normal-CON and other groups by analysis of molecular variance (AMOVA; *p* = 0.03). The abundance of *Clostridiales* was higher in the Px-CON than in the normal-CON, and its abundance was reduced by SP-M and SP-H (Figure 5C). In opposite to the *Clostridiales* ratio, the abundance of *Bacteroidales* was higher in the normal-CON and positive-CON groups than in Px-CON group, and SP-H increased its abundance but to less than that observed in the positive-CON (Figure 5C).

## 4. Discussion

Silk fibroins can be hydrolyzed by enzymes or acids, and silk fibroins hydrolyzed with a mixture of proteinases were reported to have anti-hyperglycemic activity in diabetic experimental animals [17]. However, the process is expensive and complicated, and thus, we considered that acid hydrolyzed silk fibroins might have similar anti-diabetic activities. The present study revealed that consumption of SPs alleviated hyperglycemia by enhancing insulin sensitivity and glucose-stimulated insulin secretion in an Asian type 2 diabetic animal model. The present study shows that SPs suppressed Px-induced disruption of glucose metabolism by potentiating insulin secretion and reducing insulin resistance in lean type 2 diabetic rats, and suggests SPs have potential use as anti-diabetic agents in Asian type 2 diabetes.

The cocoon shell of the silkworm contains silk fibroin fiber (70%) and a sericin layer (30%), which consists of sericin (25%) and non-sericin (5%) components [3,18]; the latter are composed of carbohydrates, salts, waxes, flavonoids, and flavonoid derivatives. The anti-hyperglycemic activities of fibroin and sericin have been studied in cells and animals [3,18]. The hydrolyses of fibroin and sericin can be achieved using enzymes or acids. Cocoons can be hydrolyzed using a mixture of proteases, but the process is difficult to control and expensive. On the other hand, acid hydrolysis using HCl may result in hydrolyzed products with similar functionalities and this technique was used to prepare that the product used in the present study. SPs contain mainly glycine and alanine, and their dipeptides and tripeptides. In the present study, these amino acids and their peptides improved symptoms in T2DM rats. Although glycine is not an essential amino acid, it is important for regulating glutathione synthesis and in the one-carbon metabolism involved in insulin resistance and DNA synthesis. Serum glycine concentrations are consistently reported to be low in obese patients and in patients with T2DM or non-alcoholic fatty liver disease. Moreover, clinical studies have suggested glycine supplementation improves the symptoms of metabolic syndrome [19]. Previous studies have also shown that Ala-His, Leu-Gly, and Pro-Pro peptides decrease serum glucose concentrations and protect against liver damage in streptozotocin-induced animal models of T2DM [20]. In our preliminary study, we found serum glycine concentrations were elevated by SP administration. A subsequent study showed that serum homocysteine concentrations (an index of insulin resistance) were reduced dose-dependently by SPs. Thus, the present study shows, glycine in SPs improved glucose metabolism.

T2DM results from an imbalance between insulin sensitivity and secretion. Unlike Westerners, insulin secretion is not increased in Asians to normalize serum glucose concentrations when insulin resistance is increased. Furthermore, even Asians with normal BMIs are susceptible to T2DM [21]. Partially pancreatectomized rats have an insulin secretion capacity of 50–60% and islet numbers at 40–50% of normal [22,23], and are not obese. Accordingly, we chose this model to represent T2DM in Asians. The present study also showed that insulin secretion capacity was 50% of normal in the Px-CON group. SPs were observed to dose-dependently reduce serum glucose concentrations in fasted and post-prandial states in Px rats to levels similar to those observed in the positive-CON group. In addition, SP treatments elevated glucose-stimulated insulin secretion dose-dependently. However, metformin failed to increase glucose-stimulated insulin secretion. Thus, our observations indicate SPs reduced hyperglycemia by potentiating glucose-stimulated insulin secretion and decreasing insulin resistance, whereas metformin decreased serum glucose levels mainly by enhancing insulin sensitivity. Previous studies have demonstrated that the consumption of silk proteins improves serum glucose concentrations mainly by improving insulin sensitivity [24,25]. However, no previous study has addressed the potentiation of glucose-stimulated insulin secretion.

Type 2 diabetes alters the gut microbiome, and host-gut microbiome interactions contribute to the efficacies of diabetic drugs [26,27]. In fact, disruption of intestinal homeostasis and associated gut microbiome dysbiosis promote T2DM [28], and intensive insulin therapy and oral hypoglycemic agents recover gut microbiome dysbiosis [28,29,30]. In one study, the *Firmicutes: Bacteroidetes* ratio was reported to be elevated in HFD-fed mice and in obese humans [27]. Our results concur with these findings, although our HFD-fed Px rats were not obese. Furthermore, we noted hyperglycemia increased the ratio of *Clostridiales* (a major species of *Firmicutes*) to *Bacteroidales* (a major species of *Bacteroidetes*), and that SP-induced reductions in hyperglycemia reduced the *Firmicutes: Bacteroidetes* ratio. Karusheva et al. have reported that short-term reduction in the intake of branched-chain amino acids lowers post-prandial insulin secretion by improving insulin sensitivity and that it enriches the *Bacteroidetes* to *Firmicutes* ratio in fecal samples [26]. Notably, the SPs used in the present study were rich in glycine and its consumption improved glucose metabolism and insulin sensitivity and beneficially normalized the gut microbiome.

## 5. Conclusions

SPs dose-dependently improved glucose regulation by potentiating insulin secretion and reducing insulin resistance in our lean T2DM rat model. Furthermore, improved glucose metabolism by SPs protected against detrimental Px-induced body composition changes and promoted gut microbiota homeostasis. SP (0.1–0.5 g acid hydrolyzed silk peptides/kg bw) was found to exhibit optimal therapeutic activity in terms of promoting glucose homeostasis in rats. In human terms, this means that about 0.04 g of acid hydrolyzed silk peptides per kg of body weight might be suitable for improving glucose homeostasis.

## Figures and Tables

**Table 1:** Energy metabolism.

	Normal-CON (*n* = 10)	Px-CON (*n* = 10)	Positive-CON (*n* = 10)	SP-L (*n* = 10)	SP-M (*n* = 10)	SP-H (*n* = 10)
Body weight gain for 8 weeks (g)	153 ± 13.1 ^a^	76.2 ± 6.8 ^d^	128 ± 13.2 ^b^	109 ± 11.3 ^c^	112 ± 10.5 ^c^	117 ± 11.2 ^b,c^
Food intake (g/day)	15.7 ± 1.4	16.2 ± 1.9	16.3 ± 1.5	17.2 ± 1.5	16.2 ± 1.9	16.2 ± 1.6
Food efficiency	0.17 ± 0.02 ^a^	0.08 ± 0.01 ^d^	0.14 ± 0.02 ^b^	0.11 ± 0.02 ^c^	0.12 ± 0.02 ^b,c^	0.13 ± 0.02 ^b^
Epididymal fat pads (g)	5.9 ± 0.7 ^a^	2.7 ± 0.4^c^	4.2 ± 0.6 ^b^	2.9 ± 0.5 ^c^	3.0 ± 0.5 ^c^	3.8 ± 0.5 ^b^
Retroperitoneal fat mass (g)	6.7 ± 0.8 ^a^	3.1 ± 0.5 ^d^	5.6 ± 0.7 ^b^	3.3 ± 0.6 ^d^	4.3 ± 0.7 ^c^	5.7 ± 0.7 ^b^
Visceral fat (g)	12.6 ± 1.5 ^a^	5.8 ± 0.8 ^d^	9.8 ± 1.2 ^b^	6.2 ± 0.9 ^d^	7.3 ± 0.9 ^c^	9.5 ± 1.2 ^b^

Food efficiency: the ratio of daily energy intake/daily weight gain. Results are expressed as means ± SDs. Pancreatectomized (Px) rats fed (1) 0.5 g dextrin/kg bw (Px-CON), (2) 0.05 g acid hydrolyzed silk peptides(SPs)/kg bw (SP-L), (3) 0.1 g SPs/kg bw (SP-M), (4) 0.5 g SPs/kg bw (SP-H), and (5) 0.04 g metformin/kg bw (positive-CON) with a high fat diet. Sham-operated rats (normal-CON) fed the same diet of Px-CON. ^a,b,c,d^ Values on the same row with different superscripts were significantly different at *p* < 0.05.

**Table 2:** Glucose metabolism during the hyperglycemic clamp.

	Normal-CON (*n* = 10)	Px-CON (*n* = 10)	Positive-CON (*n* = 10)	SP-L (*n* = 10)	SP-M (*n* = 10)	SP-H (*n* = 10)
Serum glucose at fasting state (mM)	115 ± 4.6 ^e^	291 ± 48 ^a^	142 ± 16.6 ^d^	216 ± 37 ^b^	182 ± 39 ^b,c^	172 ± 26 ^c^
Serum glucose at 2h postprandial state (mg/mL)	149 ± 4.9 ^e^	540 ± 50 ^a^	312 ± 44 ^d^	455 ± 39 ^b^	387 ± 41 ^c^	337 ± 45 ^d^
Serum insulin at fasting state (ng/mL)	2.85 ± 0.32 ^a^	1.34 ± 0.22 ^d^	1.10 ± 0.18 ^e^	2.14 ± 0.28 ^c^	2.56 ± 0.31 ^b^	1.97 ± 0.27 ^c^
Glucose infusion rates (mg/kg bw/min)	10.5 ± 1.1 ^a^	5.3 ± 0.8 ^c^	6.1 ± 1.4 ^b^	4.8 ± 0.7 ^c^	5.6 ± 0.8 ^b,c^	6.2 ± 1.0 ^b^
Insulin sensitivity at hyperglycemic state (µmol glucose · min^−1^ · 100 g^−1^ per µmol insulin/L)	29.6 ± 3.9 ^b^	20.6 ± 2.6 ^c^	36.6 ± 3.9 ^a^	22.4 ± 3.2 ^c^	21.6 ± 2.9 ^c^	27.3 ± 3.2 ^b^
Urinary glucose	-	++++	+	+++	++	+
Serum homocysteine (uM)	4.8 ± 0.8 ^d^	10.5 ± 1.7 ^a^	6.1 ± 0.8 ^c,d^	8.5 ± 1.1 ^b^	6.7 ± 0.9 ^c^	5.3 ± 0.8 ^d^

+, urinary glucose detection. Values are means ± SD. Pancreatectomized (Px) rats fed (1) 0.5 g dextrin/kg bw (Px-CON), (2) 0.05 g acid hydrolyzed silk peptides (SPs)/kg bw (SP-L), (3) 0.1 g SPs/kg bw (SP-M), (4) 0.5 g SPs/kg bw (SP-H), and (5) 0.04 g metformin/kg bw (positive-CON) with a high fat diet. Sham-operated rats (normal-CON) fed the same diet of Px-CON. ^a,b,c,d,e^ Values on the same row with different superscripts were significantly different at *p* < 0.05.

**Table 3:** The modulation of islet morphometry in the pancreas section.

	Normal-CON (*n* = 5)	Px-CON (*n* = 5)	Positive-CON (*n* = 5)	SP-L (*n* = 5)	SP-M (*n* = 5)	SP-H (*n* = 5)
β-cell area (%)	5.10 ± 0.62 ^b^	5.21 ± 0.48 ^b^	6.98 ± 0.78 ^a^	5.18 ± 0.63 ^b^	7.57 ± 0.86 ^a^	8.05 ± 0.81 ^a^
Individual β-cell size (μm^2^)	178 ± 24 ^c^	248 ± 31 ^a^	194 ± 33 ^b^	228 ± 36 ^a,b^	202 ± 28 ^b,c^	198 ± 26 ^b,c^
Pancreas weight (g)	0.46 ± 0.06 ^a^	0.18 ± 0.04 ^c^	0.21 ± 0.04 ^b,c^	0.24 ± 0.05 ^b^	0.22 ± 0.03 ^b^	0.23 ± 0.03 ^b^
Absolute β-cell mass (mg)	23.5 ± 3.4 ^a^	9.53 ± 0.95 ^e^	14.7 ± 2.4 ^c,d^	12.4 ± 1.8 ^d^	16.7 ± 2.1 ^c^	17.4 ± 2.5 ^b^
BrdU^+^ cells (% BrdU^+^ cells of islets)	0.67 ± 0.08 ^d^	0.78 ± 0.09 ^c^	0.89 ± 0.10 ^b^	0.82 ± 0.10 ^c^	0.96 ± 0.12 ^a^	0.98 ± 0.13 ^a^
Apoptosis (% apoptotic bodies of islets)	0.72 ± 0.07 ^a,b^	0.79 ± 0.09 ^a^	0.66 ± 0.08 ^b^	0.69 ± 0.07 ^b^	0.61 ± 0.08 ^b,c^	0.58 ± 0.07 ^c^

Values are means ± SD. Pancreatectomized (Px) rats fed (1) 0.5 g dextrin/kg bw (Px-CON), (2) 0.05 g acid hydrolyzed silk peptides(SPs)/kg bw (SP-L), (3) 0.1 g SPs/kg bw (SP-M), (4) 0.5 g SPs/kg bw (SP-H), and (5) 0.04 g metformin/kg bw (positive-CON) with a high fat diet. Sham-operated rats (normal-CON) fed the same diet of Px-CON. ^a,b,c,d,e^ Values on the same row with different superscripts were significantly different at *p* < 0.05.
